# Association between adverse childhood experiences and self-reported health-risk behaviors among cancer survivors: A population-based study

**DOI:** 10.1371/journal.pone.0299918

**Published:** 2024-03-21

**Authors:** Sayantani Sarkar, Brianna Jackson, Laura L. Manzo, Sangchoon Jeon, Hermine Poghosyan

**Affiliations:** 1 Yale University School of Nursing, Orange, CT, United States of America; 2 US Army, AMEDD Student Detachment, Joint Base San Antonio, Fort Sam Houston, TX, United States of America; 3 COPPER Center, Yale School of Medicine, New Haven, CT, United States of America; Wingate University, UNITED STATES

## Abstract

**Aims:**

Existing evidence shows that people who report Adverse Childhood Experiences (ACEs) are more likely to exhibit health-risk behaviors. However, limited research on this topic pertains to oncology population. We aim to address this knowledge gap by estimating the prevalence of ACEs and investigating their association with self-reported health-risk behaviors among adult cancer survivors living in the U.S.

**Methods:**

We conducted a secondary analysis using cross-sectional data from the 2021 Behavioral Risk Factor Surveillance System ACE module. We included 4,126 adults, aged ≥18 years, with a history of cancer. The outcome variable was self-reported health-risk behaviors, which included cigarette smoking, e-cigarette use, and binge alcohol drinking. Self-reported ACEs history was the primary independent variable, comprised of 11 questions regarding child abuse and dysfunctional households. We conducted descriptive statistics and multivariable logistic regression to describe the relationship between the ACE history and health-risk behaviors.

**Results:**

Overall, 84.2% of cancer survivors self-reported as White, 58.4% were women, and 76.6% were aged 65+ years. Nearly two-thirds of the sample (63.2%) self-reported at least one ACE (prior to age 18) and 21.7% engaged in ≥1 health-risk-behaviors, such as cigarette smoking, binge alcohol drinking, or e-cigarette use. Experiencing ≥3 ACEs was associated with 145% increased odds of reporting at least one health-risk behavior (OR = 2.45, 95% CI [1.78–3.38]) when compared to those without a history of ACEs. Besides, survivors who were younger, divorced, less educated, and had low income had higher odds of reporting at least one health-risk behavior.

**Conclusions:**

Overall, a history of ACEs is associated with health-risk behaviors. These all can negatively impact cancer survivors’ overall well-being. Early screening for ACE during oncologic visits can be a protective measure for preventing health-risk behaviors among cancer survivors.

## Introduction

As of January 2022, there were 18.1 million cancer survivors in the United States (U.S.), which represents more than 5% of the total population [1]. The number of cancer survivors is expected to reach 26 million by the year 2040 [1]. Cancer survivors carry a high symptom burden, including psychological distress, anxiety, depression, insomnia, and physical and functional limitations caused by cancer and cancer treatment [2–5]. Research shows that cancer survivors who experience psychological distress, anxiety, and depression are at an increased risk of engaging in different substance use behaviors, including heavy alcohol drinking and cigarette smoking [6]. A large-scale cross-sectional study reported that cancer survivors are more prone to substance use behaviors than individuals without cancer history [7]. In these circumstances, Adverse Childhood Experiences (ACEs) may serve as additional stressors and may put cancer survivors at increased risk for using substances.

Coined by clinical researchers Felitti, Anda, and colleagues (1998) in their seminal study, the term ACEs refer to a set of traumatic events or exposures that, when encountered before the age of 18 years, can lead to a toxic stress response that may negatively affect biopsychosocial development and health outcomes across the lifespan [8]. ACEs consist of ten items across three domains, which broadly assess for experiences of 1) abuse (physical, emotional, and sexual), 2) neglect (physical and emotional), and 3) household dysfunction (exposure to mental illness, incarceration, intimate partner violence, substance use, and parental separation) [9]. According to the Behavioral Risk Factor Surveillance System (BRFSS) survey data collected during the year 2011 to 2020, it is estimated that nearly 63.9% American population has experienced at least one ACE, while 17.3% have experienced three or more ACEs [10].

Existing evidence suggests that ACEs can increase the risk of several health conditions, including asthma, diabetes, myocardial infarction, coronary heart disease, stroke, depression, cancer and also lead to substance use [11–16]. Further, ACEs have been linked to at least five out of the top ten leading causes of death in the U.S. [17]. Overall, ACEs pose a threat to health and overall well-being, and this can even limit the duration and quality of one’s life [18,19]. Research suggests that ACEs are associated with poor illness experience, disrupted recovery, and decreased survival rate among cancer survivors [20]. Furthermore, ACEs are associated with high financial burden and also may lead to increased health care costs [21]. In fact, the total lifetime financial burden linked to ACEs is $124 billion in the U.S. [22].

Moreover, ACEs have been found to increase the risk of various substance use behaviors, such as smoking (cigarette and e-cigarette) and problematic alcohol drinking [11,23–26]. A study conducted among the general population showed that people with higher ACEs exposure are more likely to be a current cigarette smoker and heavy alcohol drinker compared to those who do not have any ACEs [27]. Thus, evidence suggests that preventing ACEs may reduce health-risk behaviors in adulthood, such as cigarette smoking by 33%, and problematic alcohol drinking by 24% [28]. However, there is little research pertaining to the oncology population; that is, the influence of ACEs on cancer survivors’ substance use behavior is largely unknown. A previous study of adolescent and young cancer survivors [29] revealed that traumatic events were prevalent among study participants (*n* = 92), and almost 60% experienced at least one traumatic event (encompassing traditionally-defined ACEs and medical traumatic events). However, this study did not detect any association between the traumatic events and patient-reported outcomes, including a range of physical and psychological parameters, such as cancer-specific quality of life, depression, anxiety, and generic health-related quality of life, among others [29]. Given the established link between ACEs and substance use, it is important to investigate this relationship among cancer survivors. Specifically using population-based data, this study aims to: 1) estimate the prevalence of ACEs, encompassing child abuse (physical, emotional, and sexual), and household dysfunction (exposure to mental illness, incarceration, intimate partner violence, substance use, and parental separation); 2) estimate the prevalence of three major health-risk behaviors: cigarette smoking, binge alcohol drinking and e-cigarette use; and 3) investigate the association between self-reported ACEs history and health-risk behaviors among adult cancer survivors living in 11 U.S. states

## Methods

### Study data and sample

We conducted secondary data analyses of cross-sectional data that came from the 2021 BRFSS, Adverse Childhood Experience (ACE) module were used. BRFSS is a population-based, nationally representative telephone survey of non-institutionalized adults aged ≥18 years. It is conducted every year as a collaborative effort between state health departments and Centers for Disease Control and Prevention (CDC). BRFSS was established and collected its first surveillance data on risk behaviors in 1984. In the year 2021, all 50 states the District of Columbia, Guam, Puerto Rico, and US Virgin Islands collected BRFSS data. Florida could not gather adequate BRFSS data over months to meet the minimum requirements for inclusion in the 2021 annual aggregate data set [30]. The BRFSS survey has three main parts: a) the core component, b) the optional modules, and c) state added questions that allows each state to add questions based on their needs. Each state must administer core component without any modification. Each state may choose to administer any of the optional modules without modifications or add state-specific questions to the overall questionnaire. The total sample size of BRFSS 2021 was 438,693. Overall, the survey response rate was 44% (Centers for Disease Control and Prevention, 2022b). In this study, we used data from the 2021 BRFSS ACE module that was administered by 11 U.S. states (Alabama, Arkansas, Iowa, Mississippi, Nevada, New Hampshire, North Dakota, Oregon, South Carolina, Virginia, Wisconsin).

**Fig 1:**
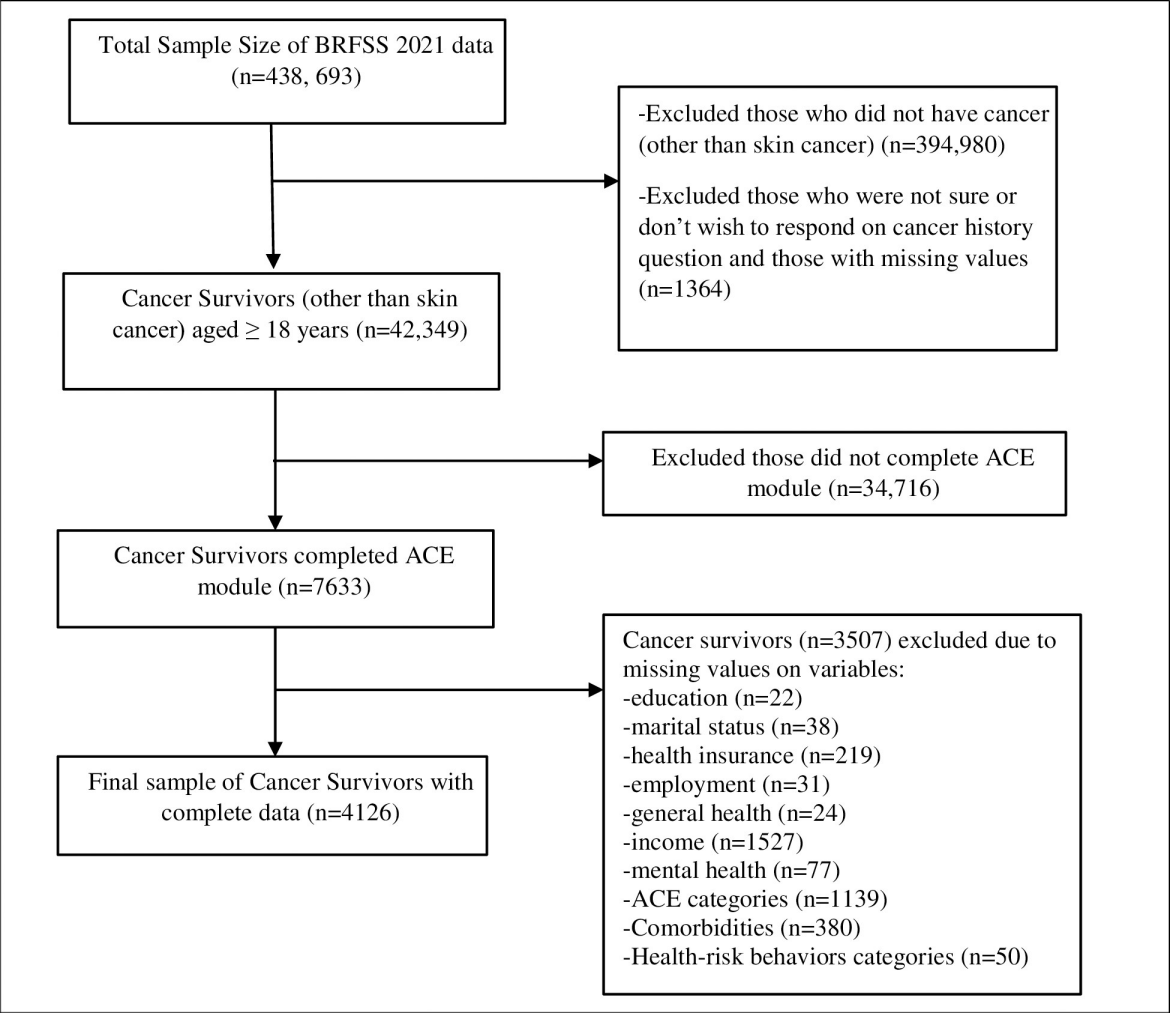
Flow chart displaying sample selection.

Respondents were defined as cancer survivors if they answered “yes” to the question: “Has a doctor, nurse, or other health professional ever told you that you had any other (than skin cancer) types of cancer?”. A total of 42,349 adult cancer survivors were identified. Then, the states that did not complete the ACE module were dropped. After eliminating this portion of the sample, 7,633 adult cancer survivors were selected. The sample was further limited to survey respondents who had complete data for all study variables. Those who responded, “don’t know/not sure,”, “refused”, or had missing values were excluded. Thus, 4126, adults with self-reported cancer history were included in the final analytic sample (Fig 1).

### Ethics approval statement

This study did not require Institutional Review Board approval because the data used is publicly available and de-identified, which does not constitute human subjects research under 45 Code of Federal Regulations part 46.102 [31].

### Measures

#### Dependent variables

The main outcome was self-reported health-risk behaviors, which include cigarette smoking status (current, non-smoker, or former), current e-cigarette use (yes, no), and binge alcohol drinking status (yes, no). Here, the term “current smokers” refers to people who have smoked at least 100 cigarettes in their lifetime, and who smoke cigarettes every day or some days [30]. “Former smokers” refers to the people who smoked at least of 100 cigarettes in their lifetime, but do not smoke at all currently [30]. By contrast, “never-smokers” are people who have not smoked a minimum of 100 cigarettes in their lifetime [30]. For our study, we combined former smokers and non-smokers together into one category. Participants who reported using e-cigarette every day and some days at the time of data collections were defined as e-cigarette users. Binge drinking is defined as men having 5 or more drinks, or women having 4 or more drinks, on a single occasion [32].We created a binary composite variable for health-risk behaviors. These were: 1) having no health-risk behaviors (respondents who self-reported as not a binge alcohol drinker, not a current e-cigarette user, and a former/never cigarette smoker), and 2) having one or more health-risk behaviors (respondents who self-reported at least one of the three health-risk behaviors: current cigarette smoking, current e-cigarette use, and/or binge alcohol drinking).

#### Independent variables

The key independent variable was a history of ACEs, which includes 11 questions regarding child abuse and household dysfunctions listed below:

1. Did you live with anyone who was depressed, mentally ill, or suicidal?
2. Did you live with anyone who was a problem drinker or alcoholic?
3. Did you live with anyone who used illegal street drugs or who abused prescription medications?
4. Did you live with anyone who served time or was sentenced to serve time in a prison, jail, or other correctional facility?
5. Were your parents separated or divorced?
6. How often did your parents or adults in your home ever slap, hit, kick, punch or beat each other up?
7. Not including spanking, (before age 18), how often did a parent or adult in your home ever hit, beat, kick, or physically hurt you in any way?
8. How often did a parent or adult in your home ever swear at you, insult you, or put you down?
9. How often did anyone at least 5 years older than you or an adult, ever touch you sexually?
10. How often did anyone at least 5 years older than you or an adult, try to make you touch them sexually?
11. How often did anyone at least 5 years older than you or an adult, force you to have sex?

For the purpose of our study, Q.1 to Q.4 questions are re-categorized as dichotomous variables (yes/no) based on their responses. For Q.5 we categorized the variable as ‘yes’ or ‘parents separated or divorced’ for respondents who indicated that their parents were separated or divorced. Similarly, respondents who self-reported that their parents were never divorced or separated and the respondents who self-reported that their parents were never married-both were combined into the ‘no’ or ‘parents not separated or divorced /parents never married’ category.

For Q.6 to Q.11, we created dichotomous variables (yes/no) based on their responses, if they reported the incident once or multiple times, we included them in the ‘yes’ category. People who never witnessed or experienced such an incident fell under the ‘no’ category. Consistent with previous studies using BRFSS data [33,34], our ACE questionnaire items are also dichotomized. Then, we combined these eleven ACE questions in to three major categories (no ACEs, 1–2 ACEs, and ≥3 ACEs). Similar stratification of ACE can be noted in the previously published articles [35].

#### Co-variates

We also included demographic factors such as age (18–34, 35–54, 55–64, ≥65), sex (female, male), race (Non-Hispanic White, Non-Hispanic Black, and other), and marital status (married, never married, divorced/separated, widowed). Socio-economic status was measured using educational attainment (high school or less, attended college, graduated), health insurance (yes/no), household annual income (<$25,000, ≥$25,000-<$50,000, ≥$50,000-<$100,000, and ≥$100,000), and residential status (rural, urban). We also included different health-related factors, such as general health status (poor-fair, good, and very good-excellent), poor mental health status in the past 30 days (0-days, 1–13 days, and ≥14 days), and comorbidity. For measuring the comorbidity, we combined nine health conditions (hypertension, high cholesterol, heart disease, asthma, arthritis, COPD, depression, kidney disease, diabetes). Then created four categories based on the number of comorbidities (no comorbidities, 1 comorbidity, 2 comorbidities, and ≥3 comorbidities).

### Statistical analysis

We implemented recommended sampling weights to generate population estimates to align with the BRFSS’s complex survey design. Here, our sample comprises 4,126 adult cancer survivors, representing 1,358,839 adult cancer survivors in the 11 U.S. states. To outline the sample characteristics, we conducted descriptive statistics of study variables, including frequencies and percentages with corresponding 95% confidence intervals (CIs). We conducted three adjusted multivariable logistic regression models focused on three health risk behavior outcomes: cigarette smoking, binge alcohol drinking, e-cigarette use, separately. Then, we created a composite variable with three health risk behaviors (cigarette smoking, binge alcohol drinking, e-cigarette use) and categorized this in to two categories (no-health risk behaviors and 1or more health risk behaviors). Subsequently, we conducted unadjusted logistic regression and also adjusted multivariable logistic regression while controlling for individual-level socio-demographic and health factors to investigate the relationship between history of ACEs and combined health-risk behaviors among cancer survivors. We reported odds ratio and 95% confidence interval. All statistical tests were conducted for two-sided test at 5% significance level using the statistical software STATA vs.17.0.

## Results

### Sample characteristics

Table 1 presents sample characteristics. Majority of the sample self-reported themselves as ≥65 years old (76.6%), Non-Hispanic White (84.2%), female (58.4%), married (62.2%), insured (98.2%), retired (47.5%), and were urban residents (86.1%). Almost two-thirds of study participants (64.5%) had either attended or graduated from college. More than half of the sample had an annual household income of $50,000 or more. Precisely 32.2% of participants self-reported having poor-fair general health, and 15.0% had poor mental health for ≥14 days in the past 30 days. Nearly half of the participants (48.2%) self-reported having ≥3 comorbidities. Overall, 13.8% of cancer survivors were current cigarette smokers, 3.2% reported e-cigarette use and 8.8% reported binge alcohol drinking. Overall, 21.7% of study participants self-reported one or more health-risk behaviors, such as cigarette smoking, binge alcohol drinking, or e-cigarette smoking.

**Table 1:** Individual-level sociodemographic, clinical, and behavioral characteristics of cancer survivors, BRFSS 2021.

	2021	
Characteristics	Unweighted sample	Weighted Percentage (95% CI)
	n = 4126	n = 1,358,839
**Age**		
18–34	55	4.64 (3.33,6.44)
35–54	151	7.28 (5.89,8.96)
55–64	318	11.46 (9.99,13.12)
≥65	3,602	76.62 (74.27,78.82)
**Sex**		
Female	2,398	58.40 (56.08,60.69)
Male	1,728	41.60 (39.31,43.92)
**Race and Ethnicity**		
Non-Hispanic White	3,635	84.23 (82.28,86.00)
Non-Hispanic Black	314	10.09 (8.75,11.62)
Other	177	5.68 (4.43,7.25)
**Marital Status**		
Never married	281	8.14 (6.73,9.83)
Married	2,364	62.21 (59.89,64.48)
Divorced/separated	634	14.51 (12.98,16.17)
Widowed	847	15.14 (13.67,16.74)
**Education**		
High-school or less	1,173	35.52 (33.28,37.82)
Attended college or technical school	1,245	35.22 (32.96,37.55)
Graduated from college or technical school	1,708	29.26 (27.44,31.15)
**Employment**		
Not in the workforce	554	19.25 (17.28,21.37)
Employed	1,101	33.21 (31.00,35.50)
Retired	2,471	47.54 (45.30,49.79)
**Income**		
<$25,000	717	18.48 (16.75,20.35)
≥ $25,000-<$50,000	1,254	30.05 (28.01,32.17)
≥$50,000-<$100,000	1,329	29.3 (27.29,31.40)
≥$100,00	826	22.17 (20.29,24.17)
**Residency**		
Rural	794	13.88 (12.58,15.29)
Urban	3,332	86.12 (84.71,87.42)
**Health Insurance**		
No	52	1.79 (1.25,2.55)
Yes	4,074	98.21 (97.45,98.75)
**General Health Status**		
Fair/Poor	1,179	31.83 (29.61,34.15)
Good	1,482	35.99 (33.85,38.20)
Excellent/Very good	1,465	32.17 (30.07,34.35)
**Poor Mental Health days**		
0-day	2,786	61.06 (58.67,63.40)
1–13 days	844	23.90 (21.85,26.08)
≥14days	496	15.04 (13.33,16.93)
**Comorbidities**		
No-comorbidity	378	10.35 (8.99,11.89)
1-comorbidity	750	18.74 (16.91,20.72)
2-comorbidities	1,001	22.7 (20.81,24.71)
≥3 comorbidities	1,997	48.21 (45.90,50.53)
**Smoking Status**		
Non-smokers/Former smokers	3,689	86.19 (84.39,87.81)
Current smokers	437	13.81 (12.19,15.61)
**Binge Drinker**		
No	3,836	91.19 (89.61,92.56)
Yes	290	8.81 (7.44,10.39)
**Current E-Cigarette User**		
No	4,049	96.8 (95.54,97.71)
Yes	77	3.20 (2.29,4.46)
**Health-risk Behaviors**		
No-health-risk behavior	3420	78.26 (76.16,80.24)
≥1-health-risk behaviors	706	21.74 (19.76,23.84)

^a^ We created health-risk variables by merging three behaviors: Cigarette smoking, binge drinking, and e-cigarette consumption. Health-risk Behavior is Categorized under two major sub-categories (no-health-risk behavior and one or more health-risk behavior).

Abbreviations: CI, Confidence Interval.

### Prevalence of ACEs

Table 2 details the prevalence of each ACE among study participants. The most reported ACE was emotional abuse (32.0%), followed by exposure to a problem drinker within the home (26.2%), physical abuse (25.2%), parental divorce (22.2%), witnessing domestic violence (18.6%), and living with someone with mental illness (17.7%). Some participants (12.9%) self-reported that they were sexually touched by an adult, or someone 5+ years older, at least once in their childhood, and nearly 10.0% self-reported that they were forced by an adult, or someone 5+ years older, to touch that person sexually at least once in their childhood. Further, 6.0% affirmed that they were forced by an adult or someone 5+ years older to have sex during childhood. Almost 10.0% of respondents self-reported having lived with someone who used drugs, and 6% reported living with someone with a history of incarceration. Overall, a little less than two third of the sample (63.2%) self-reported a minimum of one ACE. In total, 27.1% of participants self-reported having a history of 3 or more ACEs.

**Table 2:** Adverse Childhood Experiences (ACEs)^a^ among cancer survivors, BRFSS 2021.

	Questions ^b^	Sample = 4126	Weighted Percentage 95% CI
1.	**Did you live with anyone who was depressed, mentally ill, or suicidal?**		
	No	3,503	82.31 (80.34,84.11)
	Yes	623	17.69 (15.89,19.66)
2.	**Did you live with anyone who was a problem drinker or alcoholic?**		
	No	3,128	73.79 (71.64,75.84)
	Yes	998	26.21 (24.16,28.36)
3.	**Did you live with anyone who used illegal street drugs or who abused prescription medications?**		
	No	3,857	90.12 (88.38,91.62)
	Yes	269	9.88 (8.38,11.62)
4.	**Did you live with anyone who served time or was sentenced to serve time in a prison, jail, or other correctional facility?**		
	No	3,963	94.00 (92.55,95.19)
	Yes	163	6.00 (4.81,7.45)
5.	**Were your parents separated or divorced?**		
	No	3,439	77.79 (75.63,79.8)
	Yes	687	22.21 (20.2,24.37)
6.	**How often did your parents or adults in your home ever slap, hit, kick, punch or beat each other up?**		
	No	3,558	81.38 (79.22,83.37)
	Yes	568	18.62 (16.63,20.78)
7.	**Not including spanking, (before age 18), how often did a parent or adult in your home ever hit, beat, kick, or physically hurt you in any way?**		
	No	3,193	74.83 (72.67,76.87)
	Yes	933	25.17 (23.13,27.33)
8.	**How often did a parent or adult in your home ever swear at you, insult you, or put you down?**		
	No	2,926	67.97 (65.76,70.09)
	Yes	1,200	32.03 (29.91,34.24)
9.	**How often did anyone at least 5 years older than you or an adult, ever touch you sexually?**		
	No	3,661	87.06 (85.4,88.55)
	Yes	465	12.94 (11.45,14.6)
10.	**How often did anyone at least 5 years older than you or an adult, try to make you touch them sexually?**		
	No	3,798	90.47 (88.98,91.77)
	Yes	328	9.53 (8.23,11.02)
11.	**How often did anyone at least 5 years older than you or an adult, force you to have sex?**		
	No	3,943	93.97 (92.65,95.07)
	Yes	183	6.03 (4.93,7.36)
12.	**ACE-history** ^**c**^		
	No ACE	1,718	36.85 (34.74,39.01)
	1–2 ACE	1,474	36.05 (33.80, 38.37)
	≥3- ACEs	934	27.10 (24.99,29.31)

^a^ States that completed ACE modules in BRFSS 2021 are Alabama (1), Arkansas (5), Iowa (19), Mississippi (28), Nevada (32), New Hampshire (33), North Dakota (38), Oregon (41), South Carolina (45), Virginia (51), Wisconsin (55).

^b^ Each Question (1–11) is formatted as a dichotomous variable (yes/no). If the respondents reported at least one incident of ACE, it falls under the ‘yes’ category. Respondents who self-reported no ACE are included in ‘no’ category. All the missing refused, or unsure responses are coded as missing and removed from the analysis. For Question 7. There was an additional category for parents who are not married in the original BRFSS data. For creating the binary variable, we considered that under the ‘Not Separated/divorced’ category.

^C^ For ACE history: We created five sub-categories to indicate total 11 questions related to ACE. For respondents who never reported any history of ACE are considered under the No-ACE group. Similarly, people who self-reported at least 1 but below 3 ACEs falls under 1–2 ACE group and people reporting 3 or more types of ACE are under the ≥3-ACE category.

### Association between ACE and heath-risk behavior

The logistic regression model showed a statistically significant association between ACE and cigarette smoking status (S2 and S3 Tables). No statistically significant association was found between ACE and e-cigarette smoking (S6 and S7 Tables). Also, respondents self-reporting ≥3 ACEs reported higher odds of binge drinking (S4 and S5 Tables). After controlling for demographic characteristics, the logistic regression model showed 171% increased odds of reporting one or more health-risk behaviors among respondents with ≥3 ACEs (OR = 2.71, 95% CI = [1.99, 3.69]) (S1 Table).

Table 3 represents the findings of a fully adjusted logistic regression model after controlling for demographics and other health-related factors. Respondents with ≥3 ACEs had 145% increased odds of reporting at least one health-risk behavior (OR = 2.45, 95% CI = [1.78, 3.38]) than those without ACEs. Additionally, cancer survivors reporting ≥14 days of poor mental health in the past 30 days have higher odds of reporting at least one health-risk behavior compared to those without poor mental health (OR = 1.85, 95% CI = [1.31, 2.63]). Furthermore, compared to their counterparts, the odds of reporting at least one health-risk behavior was greater among respondents who were younger (18–34 years), employed, divorced/separated, those with high school or less education, with lower household income (<$25,000).

**Table 3:** Relationship between the history of ACE and prevalence of health-risk behaviors^a^ among cancer survivors, BRFSS 2021.

Characteristics	Unadjusted OR (95% CI)^b^	Adjusted OR (95% CI)^b^
ACE-history		
No-ACE	1	1
1-2-ACE	1.22 (0.91,1.65)	1.12 (0.82,1.52)
≥3ACEs	**3.52 (2.64,4.70)**	**2.45 (1.78,3.38)**
**Age**		
18–34	1	1
35–54	**0.37 (0.16,0.84)**	**0.39 (0.18,0.86)**
55–64	**0.33 (0.15,0.71)**	**0.38 (0.18,0.82)**
65+	**0.17 (0.08,0.34)**	**0.24 (0.12,0.49)**
**Sex**		
Female	1	1
Male	0.93 (0.72,1.19)	1.25 (0.95,1.64)
**Race and Ethnicity**		
Non-Hispanic White	1	1
Non-Hispanic Black	0.89 (0.61, 1.30)	0.77 (0.52,1.13)
Other	1.50 (0.82, 2.73)	0.72 (0.40,1.32)
**Marital Status**		
Never married	1	1
Married	**0.54 (0.33,0.87)**	1.39 (0.83,2.36)
Divorced/separated	1.00 (0.59,1.68)	**1.75 (1.00,3.07)**
Widowed	**0.45 (0.27,0.77)**	1.24 (0.69,2.21)
**Education**		
High-school or less	1	1
Attended college	0.80 (0.60,1.05)	0.83 (0.62,1.12)
Graduated college	0.36 (0.27,0.48)	**0.44 (0.31,0.64)**
**Employment**		
Not in a workforce	1	1
Employed	0.96 (0.69,1.34)	**1.67 (1.13,2.47)**
Retired	**0.46 (0.34,0.62)**	0.99 (0.68,1.43)
**Income**		
<$25,000	1	1
≥$25,000-<$50,000	**0.52 (0.37,0.72)**	**0.58 (0.41,0.83)**
≥$50,000-<$100,000	**0.35 (0.25,0.49)**	**0.42 (0.28,0.63)**
≥$100,00	**0.42 (0.28,0.61)**	**0.45 (0.27,0.76)**
**Residency**		
Rural	1	1
Urban	1.24 (0.93, 1.67)	1.27 (0.93,1.74)
**Health Insurance**		
No	1	1
Yes	0.66 (0.28,1.58)	1.41 (0.64,3.12)
**General Health Status**		
Fair/Poor	1	1
Good	**0.63 (0.47,0.84)**	0.89 (0.65,1.21)
Excellent/Very good	**0.63 (0.46,0.86)**	0.98 (0.67,1.42)
**Poor Mental Health Days**		
0-day	1	1
1–13 days	1.33 (0.98,1.81)	1.06 (0.76,1.48)
≥14 days	**3.39 (2.47,4.66)**	**1.85 (1.31,2.63)**
**Comorbidity**		
No-comorbidity	1	1
1-comorbidity	1.11 (0.68,1.83)	1.26 (0.78,2.01)
2-comorbidities	0.85 (0.53,1.36)	0.81 (0.50,1.31)
≥3 comorbidities	1.20 (0.78,1.84)	1.14 (0.71,1.81)

^a^ We created health-risk variables by merging three behaviors: Cigarette smoking status, binge drinking, and current e-cigarette consumption. health-risk behavior is categorized under two major sub-categories (no-health-risk behavior and one or more health-risk behaviors).

^b^ Bold numbers indicate statistical significance p < .05.

Abbreviations: CI, Confidence Interval.

## Discussion

Our study findings showed that 13.8% of cancer survivors were current smoker and 3.2% were current e-cigarette user. Another published study [36] using nationally representative data of US adults reported slightly different prevalence rates of current smoking (12.7%) and current e-cigarette use (3.8%) among cancer survivors compared to our estimation. Moreover, only 8.8% of cancer survivors self-reported being binge drinkers, a notably lower figure than that reported by Shi et al. (2023), who analyzed data from the All of Us program and found a binge drinking prevalence of 23.8% among cancer survivors [37].

To the best of our knowledge, this is the first study conducted among adult cancer survivors investigating the relationship between ACEs and three health-risk behaviors (cigarette smoking, e-cigarette smoking and binge alcohol drinking) using population-based, nationally representative data. Approximately, 63% of our sample have self-reported ACE which falls within the range provided by a recent systematic review [38]. This review reported from 40% to 95.5% prevalence rate of ACE among cancer survivors [38]. Consistent with the existing evidence [39,40], our findings also noted that emotional abuse is the most frequently reported ACE.

Overall, our study findings showed that cancer survivors who had a history of ≥3 ACEs were more likely to reported at least one health-risk behaviors. Published research featuring non-oncology populations has also noted similar associations between ACEs and binge alcohol drinking, cigarette smoking, and e-cigarette use [26,41–45]. In logistic regression models with each of the outcomes (cigarette smoking, binge alcohol drinking and e-cigarette use) separately, our study observed cancer survivors who had a history of ≥3 ACEs are more likely to report cigarette smoking and binge alcohol drinking. Consistent with our findings, another large scale study [11] with 48,526 US adults noticed higher ACE score (≥4) is associated with higher odds of several risky behaviors including smoking and binge drinking.

Future studies focused on cancer survivors are critically needed. ACEs are a source of widespread health and socioeconomic burden that necessitates urgent attention and action. For those enduring or recovering from cancer, such early adversities may intersect with the already-complex oncologic experience to influence health outcomes, quality of life, and psychosocial well-being, by way of health-risk behaviors as maladaptive coping mechanisms [46,47]. A wide body of literature have also noticed adverse oncologic outcome and diminished survival associated with the malpractice such as alcohol drinking and smoking [48–50]. Therefore, this arena requires immediate attention from the researchers and healthcare policy makers to prevent multidimensional complications associated with the past experience of ACE and substance use among cancer survivors.

In our study, we noticed younger cancer survivors have comparatively more inclination toward health-risk behaviors than their older counterparts. Our study findings are consistent with extant literature in the field of oncology [51–55]. For example, a recent study [56], used nationally representative data to compare cigarette smoking prevalence among cancer survivors with smoking-related cancers with cancer survivors with non-smoking-related cancers. They showed significantly lower odds of continued smoking among older cancer survivors as compared to their younger counterparts.

We also found an association between education and health-risk behaviors, such that those with lower educational attainment are more likely to report health-risk behaviors. This result is not unique and closely aligns with contemporary research findings in the oncology population [53,55]. A research study that investigated the smoking prevalence trend between the years 1997 to 2010 among adults (with or without childhood cancer history) found a similar association between education and smoking behavior [51]. Similarly, another cross-sectional study with cancer survivors noticed higher odds of current cigarette and e-cigarette use among comparatively less educated individuals (high school graduate & some college) than the individuals who hold graduate degree or more academic qualifications [52]. Some possible explanations may be that people with higher education often have more employment and financial security—both of which serve to mitigate stresses associated with socioeconomic status and access to resources [57]. Additionally, greater general knowledge and critical thinking skills can enhance health literacy [58], leading to a more comprehensive understanding of substance use’s harmful consequences, as well as an increased awareness of prevention services and resources [59].

Consistent with the existing literature [51,54], our findings also support the notion that cancer survivors with more disadvantaged financial status are more likely to use one or more substances. In our study, cancer survivors with higher annual household incomes were less likely to report health-risk behaviors compared to those with lower annual household incomes. We also found that compared to the cancer survivors who are not in workforce, employed cancer survivors have higher odds of having at least one health-risk behavior. On the contrary, there is substantial evidence showing unemployed cancer survivors are more likely to report health-risk behaviors [54,60]. One explanation for such different findings might be that in our study, we have combined categories like student, homemaker, people who are jobless for more or less than one year, and people who are unable to work all together under the umbrella of ‘not in workforce’. On the other hand, self-employed or waged employment categories are combined under the classification ‘employed’. Therefore, our finding needs to be considered cautiously.

In the adjusted model, our findings showed that cancer survivors who were divorced or separated were more likely to report having at least one health-risk behavior. Overall, our findings are consistent with the earlier research which analyzed the nationally representative pooled data collected during the period of seven years (2003, 2005, 2007, 2011, 2012, 2013, and 2014) and noticed that cancer survivors with divorced or separated and single conjugal status are more likely to be the current smoker [53].

### Strengths and limitations

Our study is strengthened by its large population-based and nationally representative sample of adult cancer survivors. According to the report *Evidence Gaps in Cancer Survivorship Care*: *A Report From the 2019 National Cancer Institute Cancer Survivorship Workshop* leveraging existing data resources to conduct cancer survivorship research is among the cancer survivorship research priorities [61]. Thus, future studies are needed to be conducted among cancer survivors by leveraging existing data sources.

There are several limitations to be acknowledged. The cross-sectional design of the study limits the causal inference between independent and outcome variables. Future prospective longitudinal research design can further evaluate the relationship between the history of ACE and health-risk behaviors. Several important variables such as type of cancer, onset of cancer, and treatment status are missing from our analysis which may have interference with the relationship between ACEs and health-risk behaviors. In our study, we re-categorized ACE history based on 11 out of 13 questions in the BRFSS 2021 questionnaire. Although BRFSS does not require using all questions under any module (either mandatory or optional), the elimination of these two questions may be a limitation of this study. These two questions were new additions to the BRFSS 2021 questionnaire. Many earlier studies have assessed ACE based on these 11 questions [34,35]. While defining cancer survivors, we eliminated people who only have a history of skin cancer. This attempt is similar to several prior studies using BRFSS data [62,63]. Besides, due to the secondary nature of data, we were unable to assess whether the ACE experience has an influence on the early adoption or initiation of health-risk behaviors such as cigarette smoking, binge alcohol drinking, and e-cigarette use among adult cancer survivors. This may be a new area for future investigation since studies have noticed an association between exposure to ACE and high-risk of substance use behaviors among adolescents [64,65]. Past encounters with ACE often act as a predisposing factor for various mental health disorders [66–68]. Thus, it contributes to the rising global mental health burden along with diverse substance use behaviors. Therefore, future researchers may conduct qualitative inquiry to develop more insight into the underlying psycho-social phenomenon behind the relationship between exposure to ACE and substance use behaviors.

Nearly 76% of our sample is aged 65 or older. According to the National Cancer Institute, 60% of cancer occurs among people aged 65 and above [69]. Given the increased risk of cognitive impairment or experiencing memory loss with age [70], older cancer survivors might underreport or overreport ACE experiences. Nevertheless, existing literature [71,72] has collected ACE histories from older cancer survivors. A study [73] that collected both prospective and retrospective data related to ACE revealed that self-reporting of ACE is highly dependent on individual traits and the type of ACE, such as sexual abuse, which can be under-reported. People react differently to stressors, and their capacity for forgetting and forgiving also varies. This study [73] also noted a moderate level of agreement between prospective and retrospective ACE reporting. Therefore, it is important to note that since our study relies on self-reporting, there is a possibility of reporting bias.

## Conclusions

Overall, our findings denote that ACE is associated with health-risk behaviors. Despite the adverse health effects, a significant proportion of cancer survivors report cigarette smoking, alcohol drinking, and e-cigarette consumption. Such health-risk behaviors significantly reduce the effectiveness of cancer treatment, quality of life, and survival, worsen treatment side effects, and increase the risk of cancer recurrence in the cancer population. These health-risk behaviors may also influence the occurrence of another type of cancer along with other co-morbidities. Additionally, past experiences of ACE can interfere with the psychological well-being of cancer survivors and may influence health-risk behaviors. Therefore, we strongly recommend creating more resources for ACE screening during their routine oncology visits. Such efforts may support the prevention strategies to minimize the adoption of health-risk behaviors among cancer survivors.

## Supporting information

S1 Table Relationship between the history of ACE and prevalence of health-risk behaviors among cancer survivors, BRFSS 2021. *(controlling for demographics only)*.
(DOCX)

S2 Table Relationship between the history of ACE and smoking among cancer survivors, BRFSS 2021. *(controlling for demographics only)*.
(DOCX)

S3 Table Relationship between the history of ACE and smoking among cancer survivors, BRFSS 2021. (DOCX)

S4 Table Relationship between the history of ACE and binge among cancer survivors, BRFSS 2021. *(controlling for demographics only)*.
(DOCX)

S5 Table Relationship between the history of ACE and binge among cancer survivors, BRFSS 2021. (DOCX)

S6 Table Relationship between the history of ACE and e-cigarette use among cancer survivors, BRFSS 2021. *(controlling for demographics only)*.
(DOCX)

S7 Table Relationship between the history of ACE and e-cigarette use among cancer survivors, BRFSS 2021. (DOCX)
