# Supplementary material for: Association between adverse childhood experiences and self-reported health-risk behaviors among cancer survivors: A population-based study
Source: PLoS One. 2024 Mar 21;19(3):e0299918. doi: 10.1371/journal.pone.0299918 (PMC10956880; doi:10.1371/journal.pone.0299918)
Supplement: S1 Table — (controlling for demographics only). (DOCX) [file pone.0299918.s001.docx]

**S1 Table. Relationship between the history of ACE and prevalence of health-risk behaviors among cancer survivors, BRFSS 2021. *(controlling for demographics only)***

| **Characteristics** | **Adjusted OR (95% CI)^b^** |
| --- | --- |
| **ACE-history** |  |
| No-ACE | 1 |
| 1-2 ACE | 1.17 (0.86, 1.59) |
| ≥3-ACEs | **2.71(1.99, 3.69)** |
| **Age** |  |
| 18-34 | 1 |
| 35-54 | **0.39 (0.18, 0.82)** |
| 55-64 | **0.36 (0.18, 0.75)** |
| 65+ | **0.23 (0.12, 0.45)** |
| **Sex** |  |
| Female | 1 |
| Male | 1.22 (0.94, 1.59) |
| **Race and Ethnicity** |  |
| Non-Hispanic White | 1 |
| Non-Hispanic Black | 0.78 (0.53, 1.14) |
| Other | 0.73 (0.40, 1.31) |
| **Marital Status** |  |
| Never married | 1 |
| Married | 1.38 (0.84, 2.28) |
| Divorced/separated | 1.72 (1.00, 2.96) |
| Widowed | 1.21 (0.69, **2**.12) |
| **Education** |  |
| High-school or less | 1 |
| Attended college | 0.83 (0.62, 1.11) |
| Graduated college | **0.44 (0.31, 0.63)** |
| **Employment** |  |
| Not in a workforce | 1 |
| Employed | **1.50 (1.02, 2.22)** |
| Retired | 0.89 (0.62, 1.29) |
| **Income** |  |
| <$25,000 | 1 |
| ≥$25,000-<$50,000 | **0.53 (0.37, 0.75)** |
| ≥$50,000-<$100,000 | **0.38 (0.25, 0.57)** |
| ≥$100,00 | **0.40 (0.24, 0.66)** |
| **Residency** |  |
| Rural | 1 |
| Urban | 1.33 (0.97, 1.83) |
| **Health Insurance** |  |
| No | 1 |
| Yes | 1.44 (0.66, 3.14) |

^a^ We created health-risk variables by merging three behaviors: cigarette smoking status, binge drinking, and current e-cigarette consumption. health-risk behavior is categorized under two major sub-categories (no-health-risk behavior and one or more health-risk behaviors).

^b^ Bold numbers indicate statistical significance p <.05

Abbreviations: CI, Confidence Interval.
