# Supplementary material for: Association between adverse childhood experiences and self-reported health-risk behaviors among cancer survivors: A population-based study
Source: PLoS One. 2024 Mar 21;19(3):e0299918. doi: 10.1371/journal.pone.0299918 (PMC10956880; doi:10.1371/journal.pone.0299918)
Supplement: S2 Table — (controlling for demographics only). (DOCX) [file pone.0299918.s002.docx]

**S2 Table. Relationship between the history of ACE and smoking among cancer survivors, BRFSS 2021. *(controlling for demographics only)***

| **Characteristics** | **Adjusted OR (95% CI)^b^** |
| --- | --- |
| **ACE-history** |  |
| No-ACE | 1 |
| 1-2-ACE | 1.38 (0.95, 2.01) |
| ≥3-ACEs | **3.21 (2.25, 4.58)** |
| **Age** |  |
| 18-34 | 1 |
| 35-54 | 0.52 (0.22, 1.25) |
| 55-64 | 0.52 (0.23, 1.19) |
| 65+ | **0.34 (0.16, 0.73)** |
| **Sex** |  |
| Female | 1 |
| Male | 0.96 (0.69, 1.32) |
| **Race and Ethnicity** |  |
| Non-Hispanic White | 1 |
| Non-Hispanic Black | 0.75 (0.47, 1.18) |
| Other | 1.15 (0.59, 2.25) |
| **Marital Status** |  |
| Never married | 1 |
| Married | 1.82 (0.97, 3.42) |
| Divorced/separated | **2.17 (1.10, 4.27)** |
| Widowed | **2.18 (1.09, 4.35)** |
| **Education** |  |
| High-school or less | 1 |
| Attended college | 0.74 (0.53,1.05) |
| Graduated college | **0.31 (0.21, 0.47)** |
| **Employment** |  |
| Not in a workforce | 1 |
| Employed | 1.28 (0.82, 2.00) |
| Retired | **0.63 (0.41, 0.96)** |
| **Income** |  |
| <$25,000 | 1 |
| ≥$25,000-<$50,000 | **0.54 (0.37, 0.80)** |
| ≥$50,000-<$100,000 | **0.32 (0.20, 0.51)** |
| ≥$100,00 | **0.22 (0.12, 0.41)** |
| **Residency** |  |
| Rural | 1 |
| Urban | 1.12 (0.76, 1.65) |
| **Health Insurance** |  |
| No | 1 |
| Yes | 1.08 (0.46, 2.53) |

^a^ We created health-risk variables by merging three behaviors: cigarette smoking status, binge drinking, and current e-cigarette consumption. health-risk behavior is categorized under two major sub-categories (no-health-risk behavior and one or more health-risk behaviors).

^b^ Bold numbers indicate statistical significance p<0.05

Abbreviations: CI, Confidence Interval.
