# Supplementary material for: Association between adverse childhood experiences and self-reported health-risk behaviors among cancer survivors: A population-based study
Source: PLoS One. 2024 Mar 21;19(3):e0299918. doi: 10.1371/journal.pone.0299918 (PMC10956880; doi:10.1371/journal.pone.0299918)
Supplement: S3 Table — (DOCX) [file pone.0299918.s003.docx]

**S3 Table. Relationship between the history of ACE and smoking among cancer survivors, BRFSS 2021.**

| **Characteristics** | **Adjusted OR (95% CI)^b^** |
| --- | --- |
| **ACE-history** |  |
| No-ACE | 1 |
| 1-2-ACE | 1.34 (0.91, 1.96) |
| ≥3-ACEs | **2.80 (1.92, 4.09)** |
| **Age** |  |
| 18-34 | 1 |
| 35-54 | 0.51 (0.20, 1.28) |
| 55-64 | 0.50 (0.21, 1.21) |
| 65+ | **0.32 (0.14, 0.74)** |
| **Sex** |  |
| Female | 1 |
| Male | 0.95 (0.68, 1.32) |
| **Race and Ethnicity** |  |
| Non-Hispanic White | 1 |
| Non-Hispanic Black | 0.71 (0.45, 1.13) |
| Other | 1.13 (0.57, 2.26) |
| **Marital Status** |  |
| Never married | 1 |
| Married | 1.79 (0.91, 3.52) |
| Divorced/separated | **2.15 (1.06, 4.35)** |
| Widowed | **2.24 (1.07, 4.68)** |
| **Education** |  |
| High-school or less | 1 |
| Attended college | 0.77 (0.55, 1.08) |
| Graduated college | **0.33 (0.22, 0.50)** |
| **Employment** |  |
| Not in a workforce | 1 |
| Employed | **1.60 (1.02, 2.51)** |
| Retired | 0.70 (0.46, 1.08) |
| **Income** |  |
| <$25,000 | 1 |
| ≥$25,000-<$50,000 | **0.64 (0.43, 0.96)** |
| ≥$50,000-<$100,000 | **0.40 (0.25, 0.64)** |
| ≥$100,00 | **0.28 (0.15, 0.54)** |
| **Residency** |  |
| Rural | 1 |
| Urban | 1.09 (0.75, 1.59) |
| **Health Insurance** |  |
| No | 1 |
| Yes | 1.07 (0.45, 2.55) |
| **General Health Status** |  |
| Fair/Poor | 1 |
| Good | 0.74 (0.53, 1.04) |
| Excellent/Very good | **0.58 (0.37, 0.92)** |
| **Poor Mental Health Days** |  |
| 0-day | 1 |
| 1-13 days | 0.85 (0.57, 1.25) |
| ≥14 days | **1.69 (1.12, 2.54)** |
| **Comorbidity** |  |
| No-comorbidity | 1 |
| 1-comorbidity | 1.32 (0.65, 2.67) |
| 2-comorbidities | 0.92 (0.47, 1.83) |
| ≥3 comorbidities | 1.40 (0.73, 2.66) |

^a^ We created health-risk variables by merging three behaviors: cigarette smoking status, binge drinking, and current e-cigarette consumption. health-risk behavior is categorized under two major sub-categories (no-health-risk behavior and one or more health-risk behaviors).

^b^ Bold numbers indicate statistical significance p <.05

Abbreviations: CI, Confidence Interval.
