# Supplementary material for: Association between adverse childhood experiences and self-reported health-risk behaviors among cancer survivors: A population-based study
Source: PLoS One. 2024 Mar 21;19(3):e0299918. doi: 10.1371/journal.pone.0299918 (PMC10956880; doi:10.1371/journal.pone.0299918)
Supplement: S4 Table — (controlling for demographics only). (DOCX) [file pone.0299918.s004.docx]

**S4 Table. Relationship between the history of ACE and binge among cancer survivors, BRFSS 2021. *(controlling for demographics only)***

| **Characteristics** | **Adjusted OR (95% CI)^b^** |
| --- | --- |
| **ACE-history** |  |
| No-ACE | 1 |
| 1-2ACE | 1.26 (0.80, 1.98) |
| 3+ACE | **2.62 (1.66, 4.13)** |
| **Age** |  |
| 18-34 | 1 |
| 35-54 | **0.29 (0.12, 0.73)** |
| 55-64 | **0.29 (0.12, 0.72)** |
| 65+ | **0.24 (0.11, 0.55)** |
| **Sex** |  |
| Female | 1 |
| Male | **1.94 (1.33, 2.83)** |
| **Race and Ethnicity** |  |
| Non-Hispanic White | 1 |
| Non-Hispanic Black | 0.98 (0.53,1.80) |
| Other | 0.67 (0.28,1.59) |
| **Marital Status** |  |
| Never married | 1 |
| Married | 1.01 (0.51, 2.00) |
| Divorced/separated | 1.45 (0.67, 3.15) |
| Widowed | 0.78 (0.30, 2.03) |
| **Education** |  |
| High-school or less | 1 |
| Attended college | 1.02 (0.66, 1.56) |
| Graduated college | 0.63 (0.39, 1.02) |
| **Employment** |  |
| Not in a workforce | 1 |
| Employed | **2.98 (1.63, 5.45)** |
| Retired | 1.84 (0.95, 3.57) |
| **Income** |  |
| <$25,000 | 1 |
| ≥$25,000-<$50,000 | 0.93 (0.51, 1.68) |
| ≥$50,000-<$100,000 | 0.98 (0.53, 1.84) |
| ≥$100,00 | 1.20 (0.58, 2.48) |
| **Residency** |  |
| Rural | 1 |
| Urban | 1.44 (0.89, 2.35) |
| **Health Insurance** |  |
| No | 1 |
| Yes | 0.79 (0.26, 2.40) |

^a^ We created health-risk variables by merging three behaviors: cigarette smoking status, binge drinking, and current e-cigarette consumption. health-risk behavior is categorized under two major sub-categories (no-health-risk behavior and one or more health-risk behaviors).

^b^ Bold numbers indicate statistical significance p <.05

Abbreviations: CI, Confidence Interval.
