# Supplementary material for: Association between adverse childhood experiences and self-reported health-risk behaviors among cancer survivors: A population-based study
Source: PLoS One. 2024 Mar 21;19(3):e0299918. doi: 10.1371/journal.pone.0299918 (PMC10956880; doi:10.1371/journal.pone.0299918)
Supplement: S5 Table — (DOCX) [file pone.0299918.s005.docx]

**S5 Table. Relationship between the history of ACE and binge among cancer survivors, BRFSS 2021.**

| **Characteristics** | **Adjusted OR (95% CI)^b^** |
| --- | --- |
| **ACE-history** |  |
| No-ACE | 1 |
| 1-2-ACE | 1.18 (0.75, 1.86) |
| 3+ACE | **2.44 (1.53, 3.89)** |
| **Age** |  |
| 18-34 | 1 |
| 35-54 | **0.31 (0.12, 0.77)** |
| 55-64 | **0.32 (0.13, 0.81)** |
| 65+ | **0.29 (0.13, 0.68)** |
| **Sex** |  |
| Female | 1 |
| Male | **2.03 (1.40, 2.96)** |
| **Race and Ethnicity** |  |
| Non-Hispanic White | 1 |
| Non-Hispanic Black | 1.05 (0.57, 1.94) |
| Other | 0.70 (0.31, 1.59) |
| **Marital Status** |  |
| Never married | 1 |
| Married | 1.07 (0.52, 2.20) |
| Divorced/separated | 1.55 (0.69, 3.49) |
| Widowed | 0.82 (0.31, 2.19) |
| **Education** |  |
| High-school or less | 1 |
| Attended college | 1.02 (0.66, 1.57) |
| Graduated college | **0.59 (0.37, 0.96)** |
| **Employment** |  |
| Not in a workforce | 1 |
| Employed | **3.05 (1.63, 5.71)** |
| Retired | **2.00 (1.02, 3.93)** |
| **Income** |  |
| <$25,000 | 1 |
| ≥$25,000-<$50,000 | 0.98 (0.56, 1.73) |
| ≥$50,000-<$100,000 | 0.98 (0.53, 1.82) |
| ≥$100,00 | 1.21 (0.59, 2.46) |
| **Residency** |  |
| Rural | 1 |
| Urban | 1.42 (0.85, 2.37) |
| **Health Insurance** |  |
| No | 1 |
| Yes | 0.72 (0.22, 2.33) |
| **General Health Status** |  |
| Fair/Poor | 1 |
| Good | 0.93 (0.56, 1.55) |
| Excellent/Very good | 1.52 (0.84, 2.74) |
| **Poor Mental Health Days** |  |
| 0-day | 1 |
| 1-13 days | 1.28 (0.79, 2.09) |
| ≥14 days | **1.87 (1.11, 3.14)** |
| **Comorbidity** |  |
| No-comorbidity | 1 |
| 1-comorbidity | 0.78 (0.42, 1.45) |
| 2-comorbidities | 0.70 (0.38, 1.28) |
| ≥3 comorbidities | 0.74 (0.40, 1.38) |

^a^ We created health-risk variables by merging three behaviors: cigarette smoking status, binge drinking, and current e-cigarette consumption. health-risk behavior is categorized under two major sub-categories (no-health-risk behavior and one or more health-risk behaviors).

^b^ Bold numbers indicate statistical significance p <.05

Abbreviations: CI, Confidence Interval.
