# Supplementary material for: Association between adverse childhood experiences and self-reported health-risk behaviors among cancer survivors: A population-based study
Source: PLoS One. 2024 Mar 21;19(3):e0299918. doi: 10.1371/journal.pone.0299918 (PMC10956880; doi:10.1371/journal.pone.0299918)
Supplement: S6 Table — (controlling for demographics only). (DOCX) [file pone.0299918.s006.docx]

**S6 Table. Relationship between the history of ACE and e-cigarette use among cancer survivors, BRFSS 2021. *(controlling for demographics only)***

| **Characteristics** | **Adjusted OR (95% CI)^b^** |
| --- | --- |
| **ACE-history** |  |
| No-ACE | 1 |
| 1-2ACE | 0.49 (0.20,1.22) |
| 3+ACE | 1.79 (0.82, 3.91) |
| **Age** |  |
| 18-34 | 1 |
| 35-54 | 0.39 (0.13, 1.17) |
| 55-64 | **0.25 (0.09, 0.71)** |
| 65+ | **0.07 (0.02, 0.20)** |
| **Sex** |  |
| Female | 1 |
| Male | 1.92 (0.94, 3.96) |
| **Race and Ethnicity** |  |
| Non-Hispanic White | 1 |
| Non-Hispanic Black | 0.89 (0.28, 2.88) |
| Other | 0.60 (0.20,1.76) |
| **Marital Status** |  |
| Never married | 1 |
| Married | 0.91 (0.38, 2.20) |
| Divorced/separated | 1.15 (0.43, 3.11) |
| Widowed | 0.37 (0.11,1.20) |
| **Education** |  |
| High-school or less | 1 |
| Attended college | 2.02 (0.97, 4.21) |
| Graduated college | 1.08 (0.33, 3.55) |
| **Employment** |  |
| Not in a workforce | 1 |
| Employed | 2.12 (0.95, 4.71) |
| Retired | 2.16 (0.69, 6.79) |
| **Income** |  |
| <$25,000 | 1 |
| ≥$25,000-<$50,000 | **0.19 (0.07, 0.50)** |
| ≥$50,000-<$100,000 | **0.18 (0.05, 0.58)** |
| ≥$100,00 | **0.18 (0.04, 0.74)** |
| **Residency** |  |
| Rural | 1 |
| Urban | 0.67 (0.34,1.30) |
| **Health Insurance** |  |
| No | 1 |
| Yes | 1.60 (0.31, 8.21) |

^a^ We created health-risk variables by merging three behaviors: cigarette smoking status, binge drinking, and current e-cigarette consumption. health-risk behavior is categorized under two major sub-categories (no-health-risk behavior and one or more health-risk behaviors).

^b^ Bold numbers indicate statistical significance p <0.05

Abbreviations: CI, Confidence Interval.
