# Supplementary material for: Association between adverse childhood experiences and self-reported health-risk behaviors among cancer survivors: A population-based study
Source: PLoS One. 2024 Mar 21;19(3):e0299918. doi: 10.1371/journal.pone.0299918 (PMC10956880; doi:10.1371/journal.pone.0299918)
Supplement: S7 Table — (DOCX) [file pone.0299918.s007.docx]

**S7 Table. Relationship between the history of ACE and e-cigarette use among cancer survivors, BRFSS 2021.**

| **Characteristics** | **Adjusted OR (95% CI)^b^** |
| --- | --- |
| **ACE-history** |  |
| No-ACE | 1 |
| 1-2-ACE | 0.47 (0.20, 1.10) |
| 3+ACE | 1.64 (0.77, 3.48) |
| **Age** |  |
| 18-34 | 1 |
| 35-54 | 0.38 (0.13, 1.14) |
| 55-64 | **0.25 (0.08, 0.76)** |
| 65+ | **0.07 (0.02, 0.21)** |
| **Sex** |  |
| Female | 1 |
| Male | **2.10 (1.08, 4.11)** |
| **Race and Ethnicity** |  |
| Non-Hispanic White | 1 |
| Non-Hispanic Black | 0.88 (0.28, 2.76) |
| Other | 0.52 (0.17, 1.56) |
| **Marital Status** |  |
| Never married | 1 |
| Married | 0.88 (0.36, 2.14) |
| Divorced/separated | 1.14 (0.43, 3.04) |
| Widowed | 0.36 (0.11, 1.14) |
| **Education** |  |
| High-school or less | 1 |
| Attended college | 2.08 (0.99, 4.35) |
| Graduated college | 1.14 (0.34, 3.84) |
| **Employment** |  |
| Not in a workforce | 1 |
| Employed | **2.33 (1.03, 5.27)** |
| Retired | 2.25 (0.72, 7.01) |
| **Income** |  |
| <$25,000 | 1 |
| ≥$25,000-<$50,000 | **0.22 (0.08, 0.60)** |
| ≥$50,000-<$100,000 | **0.21 (0.07, 0.68)** |
| ≥$100,00 | **0.21 (0.05, 0.90)** |
| **Residency** |  |
| Rural | 1 |
| Urban | 0.61 (0.32, 1.19) |
| **Health Insurance** |  |
| No | 1 |
| Yes | 1.55 (0.33, 7.33) |
| **General Health Status** |  |
| Fair/Poor | 1 |
| Good | 1.11 (0.52, 2.40) |
| Excellent/Very good | 0.84 (0.30, 2.36) |
| **Poor Mental Health Days** |  |
| 0-day | 1 |
| 1-13 days | 1.20 (0.58, 2.46) |
| ≥14 days | 1.48 (0.61, 3.60) |
| **Comorbidity** |  |
| No-comorbidity | 1 |
| 1-comorbidity | 2.06 (0.49, 8.58) |
| 2-comorbidities | 1.70 (0.42, 6.85) |
| ≥3 comorbidities | 2.09 (0.51, 8.58) |

^a^ We created health-risk variables by merging three behaviors: cigarette smoking status, binge drinking, and current e-cigarette consumption. health-risk behavior is categorized under two major sub-categories (no-health-risk behavior and one or more health-risk behaviors).

^b^ Bold numbers indicate statistical significance p <.05

Abbreviations: CI, Confidence Interval.
